# The Acaricidal Activity of Venom from the Jellyfish *Nemopilema nomurai* against the Carmine Spider Mite *Tetranychus cinnabarinus*

**DOI:** 10.3390/toxins8060179

**Published:** 2016-06-09

**Authors:** Huahua Yu, Yang Yue, Xiangli Dong, Rongfeng Li, Pengcheng Li

**Affiliations:** 1 Key Laboratory of Experimental Marine Biology, Institute of Oceanology, Chinese Academy of Sciences, 7 Nanhai Road, Qingdao 266071, China; yuhuahua@qdio.ac.cn (H.Y.); buqun08@163.com; (Y.Y.); rongfengli@qdio.ac.cn (R.L.); 2 College of Earth Sciences, University of Chinese Academy of Sciences, 19 Yuquan Road, Beijing 100039, China; 3 Institute of Agriculture Sciences and Plant Protect, Qingdao Agriculture University, Chengyang, Qingdao 266109, China; xldong0326@163.com

**Keywords:** acaricidal activity, jellyfish, *Nemopilema nomurai*, venom

## Abstract

The carmine spider mite *Tetranychus cinnabarinus* (*T. cinnabarinus*) is a common polyphagous pest that attacks crops, vegetables, flowers, and so on. It is necessary to find lead compounds for developing novel, powerful, and environmentally-friendly acaricides as an alternative approach to controlling the carmine spider mite because of the serious resistance and residual agrochemicals in the environment. In addition, the study on the acaricidal activities of marine bioactive substances is comparatively deficient. In the present study, the acaricidal activity of venom (NnFV) from the jellyfish *Nemopilema nomurai* against the carmine spider mite *T. cinnabarinus* was determined for the first time. The venom had contact toxicity, and the 24-h LC_50_-value was 29.1 μg/mL. The mite body wall was affected by the venom, with the mite body having no luster and being seriously shrunken after 24 h. *T. cinnabarinus* was a potential target pest of NnFV, which had potential as a type of natural bioacaricide. The repellent activity and systemic toxicity of the venom against *T. cinnabarinus* were also studied. However, NnFV had no repellent activity and systemic toxicity against *T. cinnabarinus*.

## 1. Introduction

The carmine spider mite *T*. *cinnabarinus* (Boisduval) (Acarina: Tetranychidae) is a polyphagous pest that attacks crops, vegetables, and flowers grown in fields and greenhouses worldwide [1]. It usually feeds on leaves whose epidermis is damaged, resulting in yellow and brown blotches accompanied by dry leaf-fall. Reduction in both the quality and quantity of the crops results from severe mite-feeding. It is recognized as the most difficult mite to control due to its small size, short generation time, strong adaptability, and ability to produce resistance. Presently, the widely practiced management program to control the carmine spider mite is chemical control by using pyridaben, azacyclotin, amitraz, and other classes of acaricides. However, resistance has emerged, and residual agrochemicals in the environment have become serious [2]. Therefore, it is necessary to find lead compounds for developing novel, powerful, and environmentally-friendly acaricides, as an alternative approach to controlling the carmine spider mite.

A multitude of acaricidal substances from plants such as *Phytolacca americana* L., *Origanum onites* L., *Thymbra spicata* L. subsp. *spicata,* *Mentha spicata* L., *Aloe vera* L., *Artemisia annua* L., and *Piper longum* L. have already been reported. They consisted of a broad range of compounds including esculentoside P, carvacrol, thyme, oregano, oleanolic acid, and so on [1,2,3,4,5,6]. 2,4-di-tert-butylphenol and ethyl oleate from the extract of the plant *C. camphora* extract had acaricidal activity against *T*. *cinnabarinus*, and the LC_50_-values were 855.83 and 1011.84 mg/kg in slide-dip tests, respectively. The acetone extract from *Aloe vera* L. leaves possessed acaricidal activity with a LC_50_-value of 90 mg/kg at 72 h after treatment in a slide-dip test. However, the study on the acaricidal activities of marine bioactive substances was comparatively incomplete.

The main component of jellyfish venom is protein [7]. Jellyfish venom has been proven to have many bioactivities such as enzymatic, hemolytic, insecticidal, and antioxidant activities [8,9,10,11,12,13]. Cardiac toxicity and even lethal effect have also been described using *in vivo* models [14,15,16]. However, the acaricidal activity of venom from jellyfish had never been studied. *Nemopilema nomurai* Uchida (*N. nomura*), 1936, also named *Stomolophus meleagris* L. Agassiz, 1862 [17], is one of the most toxic giant jellyfishes. It has bloomed increasingly in the Southern Yellow Sea and the northern part of the East China Sea, and its distribution has a relationship with the water current and possible predators [18]. Only a small portion of *N. nomurai* specimens is processed for food due to envenomation and its high water content (approximately 95%).

In an attempt to develop a new eco-friendly tool as an alternative to a conventional acaricide and an approach to utilizing jellyfish with a high value, the present study aims to assay the acaricidal activity of the venom from the tentacle of jellyfish *N. nomurai* against the carmine spider mite *T. cinnabarinus*.

## 2. Results and Discussion

### 2.1. Acaricidal Activity of NnFV against T. cinnabarinus

Table 1 shows the contact toxicity of NnFV against *T. cinnabarinus.* The NnFV had stronger acaricidal activity against *T. cinnabarinus* than Dicofol, and the toxic effect was dose-dependent. The LC_50_-value of NnFV was 29.1 μg/mL, whereas that of Dicofol was 1350.9 μg/mL. In the regression equation, *b* is the slope and denotes the different degrees of sensitivity against the mites. A larger *b*-value indicates more consistency of the acaricides against the mites. The *b*-values of NnFV and Dicofol were 1.44 and 3.22, respectively, indicating that the sensitivity of *T. cinnabarinus* to NnFV was significantly different individually than that of *T. cinnabarinus* to Dicofol.

Natural bioactive compounds were found to be a promising alternative for mite control, and more attention had been paid to the acaricidal activities of extracts from plants against the mites. Ding *et al.* [3] studied the acaricidal activities of different extracts from *Phytolacca americana* L. The LC_50_-value of the root acetone extract against adult female mites was 2.16 mg/mL after 48 h of treatment. The LC_50_-values of the ninth fraction from the acetone extract were 1.26, 0.20, and 0.10 mg/mL after 24, 48, and 72 h of treatment, respectively [3]. The acaricidal activities of six different extracts (hexane, chloroform, ether, ethyl acetate, 70% ethanol, and water) from *Syzygium cumini* L. against the mite *T. urticae* were studied by Shalaby *et al.* [6]. The LC_50_-values were 101, 118, 102, 98, 85, and 120 μg/mL after 24 h of treatment, respectively [6]. Thus, the acaricidal activity of NnFV was stronger than the extracts from the plants *Phytolacca Americana* L. and *Syzygium cumini* L. However, essential oils from medicinal plants had stronger acaricidal activities than NnFV, and the LC_50_-values of essential oils from *T. s. spicata*, *O. onites*, *M. spicata*, and *L. s. stoechas* against *T. cinnabarinus* were 0.53, 0.69, 1.83, and 2.92 μg/mL, respectively [1].

Marine substances have many bioactivities and have been widely applied in health, materials, and agriculture. More attention has been paid to the antibacterial activities including human-pathogen, food-pathogen, aqua-pathogen, and plant-pathogen [19,20,21,22]. However, the acaricidal activities of extracts from marine organisms have been less studied to our knowledge, and only two papers about the acaricidal activities of the extracts from marine organisms could be retrieved at present. NnFV had a stronger acaricidal activity than the extracts from marine mangrove plants, and the LC_50_-values of extracts from *Cerbera manghas* L., *Myoporum bontioides* A. Gray, *Clerodendrum inerme* (L.) Gaertn, *Hibiscustiliaceus* L., and *Lumnitzera racemosa* Willd were 1.84, 2.88, 2.92, 4.93, and 6.57 g/L, respectively [23]. However, the extracts of *n*-hexane and dichloromethane from *Laurencia dentroidea* (Rhodophyta) and *Canistrocarpus cervicornis* (Phaeophyta) had high acaricidal activities against *Tetranychus urticae* and their LC_50_-values were 0.05 and 0.07 μg/cm^2^, respectively [24].

Venomous animals produce diverse chemical cocktails that are used for defense, prey capture, competitor deterrence, and/or extraoral digestion [25]. These venoms have proven to be a valuable source of pharmacologically active compounds. The reports about the potential application of venom in agriculture have mainly focused on the insecticidal activities of spider and scorpion venom [26,27,28]. We reported that the venom from the jellyfish *Rhopilema esculentum* had insecticidal activity against the *Stephanitis pyri* Fabricius, *Aphis medicaginis* Koch, and *Myzus persicae* Sulzer, with 48 h LC_50_-values of 123.1, 581.6, and 716.3 μg/mL, respectively [11]. The acaricidal activity of venom from the jellyfish *N. nomurai* against the *T. cinnabarinus* was studied for the first time, and it was higher than the insecticidal activity of venom from the jellyfish *Rhopilema esculentum* against the *Stephanitis pyri* Fabricius, *Aphis medicaginis* Koch, and *Myzus persicae* Sulzer.

In the present experiment, the body wall color of treated mites was deepened, the body size was decreased slightly, and the vigor decreased significantly after 1 h. The treated mites were dead, and the body wall was dehydrated and shrunken after 8 h. The body wall of treated mites had no luster and was significantly shrunken after 24 h. Meanwhile, the color, shape, and vitality of the control group had almost no change after 24 h. These results suggested that NnFV affected the body wall of mites, possibly resulting in the death of mites.

### 2.2. Repellent Activity of NnFV against T. cinnabarinus

**Figure 1:**
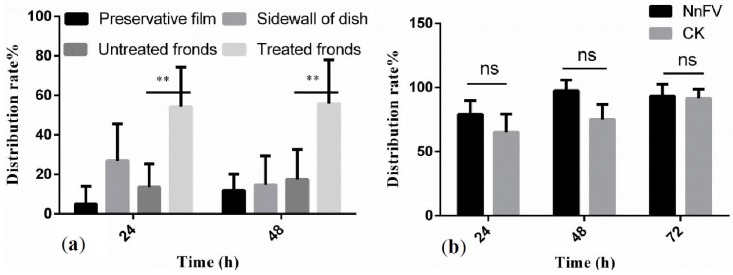
Repellent activity of NnFV against *T. cinnabarinus.* (**a**) Distribution rate of adult female mites in the leaf-dip assays. (**b**) Distribution rate of adult female mites in the potted seeding assays. Distribution rate% = Ns × 100/Nt; Ns: Number of mites on the treated host; Nt: Total number of mites employed in the experiment; ** *p* < 0.01 *vs*. untreated fronds; ns: not significant, *n* = 3.

Figure 1 shows the results of the repellent activity of NnFV against the *T. cinnabarinus* at 29 μg/mL (LC_50_ of NnFV, 95% confidence interval for LC_50_ was 22.0–38.5 μg/mL). In leaf-dip assays, the distribution rate of adult female mites on the treated fronds was higher than that on untreated fronds at 24 and 48 h. In potted seeding assays, the distribution rate of adult female mites on the treated host had no significant difference with that on the control peanut (CK) at 24, 48, and 72 h. Thus, NnFV had no repellent activity against *T. cinnabarinus.*

The modes of action of acaricides include contact toxicity, repellent activity, systemic toxicity, and stomach toxicity. The repellent activity is related to the components of acaricides, especially the volatile constituents with aromatic odors [29]. Phenolic compounds with aromatic odors such as Sculentoside P, essential oil, and eugenic acid had repellent activities. Azaizeh reported that 16 aromatic plant extracts of local species showed significant repellent activities [30]. In addition, extracts from plants such as asarum, *Platycladus orientalis* (L.) France, and *Euphorbia australis* L. typically had aromatic odors and had repellent activities against *T. urticae* and *T. cinnabarinus,* respectively [29,31]. The main component of NnFV was protein without an aromatic odor, possibly resulting in no repellent activity.

### 2.3. Systemic Toxicity of NnFV against T. cinnabarinus

**Figure 2:**
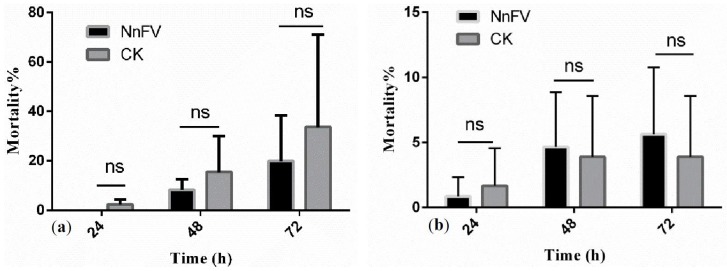
Systemic toxicity of NnFV against *T. cinnabarinus.* (**a**) Mortality of adult female mites in the petiole-dip assays. (**b**) Mortality of adult female mites in the root-dip assays. ns: not significant, *n* = 3.

Figure 2 shows the systemic toxicity of NnFV against *T. cinnabarinus*. In petiole-dip and root-dip assays, the mortalities at 24, 48, and 72 h were all below 20% at 255 μg/mL (approximately 10 times the LC_50_ of NnFV, 95% confidence interval for LC_50_ was 22.0–38.5 μg/mL), indicating that NnFV had almost no systemic toxicity against *T. cinnabarinus*.

The absorbance and conduction of the components in acaricides are necessary for systemic toxicity. In the petiole-dip assays, the components are subjected to the barrier of wax in the stratum corneum to enter into the plant. The stratum corneum of petiole is thick, and lipid is the main component of stratum corneum. Therefore, the lipophilic component enters into the plant easily. In the root-dip assays, the components must diffuse transmembrane to enter into the xylem and conduct upward, and this process depends on the lipophilicity and concentration difference of the components. For the acaricide, it is a transmembrane diffusion and upward conduction process of the component from the root, to the petiole, and finally to the surface of the plant leaves. Components that could be conducted upward must be lipophilic independent of the type of plant and metabolic physiology. The upward conduction properties of the lipophilic components were closely related to the type and absorption properties of the plants. A component could exhibit different degrees of conduction on one or more plants independent of the molecular structure of the component [32]. Four different extracts from *Stellera chamaejasme* root had systemic toxicity against *T. viennensisi.* Of the four extracts, chloroform and petroleum ether extracts had higher systemic toxicities than alcohol and water extracts [33]. It was clear that the lipophilic components in the chloroform and petroleum ether extracts were greater than those in the alcohol and water extracts, consistent with the finding that lipophilic components could be conducted upward. Squalene and scopoletin, the lipophilic components from plant *Stellera chamaejasme* also had systemic toxicity against *T. cinnabarinus* [34,35]. The main component of NnFV was hydrophilic proteins with molecular weights of 20–100 kDa [36], possibly resulting in no systemic toxicity.

## 3. Conclusions

NnFV was extracted from the jellyfish ecthoreum, which was discarded because of envenomation and toxicity. As a type of marine bioactive substance, NnFV had contact toxicity against the carmine spider mite *T. cinnabarinus*, and the 24-h LC_50_-value was 29.1 μg/mL. NnFV affected the mite body wall, with the mites having no luster and being seriously shrunken after 24 h. *T. cinnabarinus* was a potential target pest of NnFV. In addition, the venom from the jellyfish *N. nomurai* exhibited acceptable environmental safety because of no toxicity against the non-target insect the silkworm [37]. Therefore, NnFV could be eligible as a type of natural bioacaricide. The present study provided a potential way of developing a bioacaricide while simultaneously improving the value of jellyfish. Further studies are indeed recommended to better define the components, the mechanism of action of NnFV, and the real feasibility of NnFV as a bioacaricide.

## 4. Materials and Methods 

### 4.1. Biological Material 

The carmine spider adult female mites were used to assay the acaricidal activity of jellyfish venom. A laboratory stock culture of *T. cinnabarinus* was maintained in the laboratory for 5 years with no acaricide exposure. They were reared on peanut seedlings in a climate chamber (Jiangnan Instrument Factory, Ningbo, China) at 26 °C, 60%–65% relative humidity (RH), and a light-to-dark ratio (L:D) of 16:8 h.

### 4.2. Venom Preparation

The frequent bloom of jellyfish has seriously affected tourism, fishing, military affairs, and marine sport events. The majority of jellyfish in bloom is the *N. nomurai* specimen in China. Therefore, jellyfish could be collected in sufficient amount, and fishing for jellyfish is permitted by the department of fisheries in China. *N. nomurai* specimens were collected in the Aoshan Bay in Qingdao, Shandong province, China, in August 2012. The ecthoreum were manually excised *in vivo*, packed in polythene bags, and frozen immediately at −20 °C. The frozen ecthoreum were then sonicated in a cold (4 °C) phosphate buffer solution (0.01 mol/L, pH 6) eight times for 30 s, each time at 100 mV. The resultant fluids were clarified by centrifugation (15,000 *g*) for 20 min at 4 °C and used as full venom (NnFV) for that the resultant fluids contained tentacle venom and nematocyst venom. The concentration of the NnFV was determined by the Bradford method, using bovine serum albumin as a standard [38].

### 4.3. Acaricidal Activity Bioassays

The FAO-recommended slide-dip method was used to evaluate the acaricidal activity [39]. *T. cinnabarinus* adult females were picked up with a fine brush, and their backs were stuck tightly on double-sided adhesive tape (1 cm × 2 cm) on a slide, with 20 mites on each slide. One hour later, the mites were examined under a binocular microscope (4×) (Olympus Corporation, Tokyo, Japan) to remove any dead, unanimated, or improperly positioned mites. Then, the number of mites on the slide was replenished to 20 with vital ones. The slides were dipped in the NnFV solutions, shaken gently for 5 s, and then removed. The excess solution was immediately absorbed with filter paper. Then, the mites were cultured at 26 ± 1 °C, 60%–65% RH, and a photoperiod of 16:8 h (L:D). The slide-dip test involved three replicates at the five NnFV dosages (481, 96, 24, 12, and 6 μg/mL), with the phosphate buffer solution (0.01 mol/L, pH 6) used as a control. Dicofol (Sinopharm Chemical Regent Co., Ltd, Shanghai, China) at five dosages (5000, 2500, 1250, 625, and 312.5 μg/mL) was used as a positive control. The poisoning symptoms were examined under a microscope at 1, 4, 8, and 24 h after treatment. Mites were considered dead if the appendages did not respond to a touch with a fine brush, and mortality was recorded at 24 h after treatment. Mortality was corrected by Abbot’s formula [40]:

(1) $$Corrected\;mortality=\frac{\%\;test\;mortality-\%\;control\;mortality}{100-\%\;control\;mortality}\times 100\;$$

### 4.4. Repellent Activity of NnFV against T. cinnabarinus

Leaf-dip assays: The petioles of young peanut fronds were moistened with wet filter paper, and the young fronds were then dipped in the NnFV solutions for 10 s, taken out, dried, and then laid on the side of the dish (Φ 15 cm) bottom covered with three layers of wet filter paper. Another set of young fronds were immersed in water for 10 s, taken out, dried, and then symmetrically laid on the other side of the dish (Φ 15 cm). Thirty adult female mites were placed on the middle of the filter paper, and the dish was then covered with a pricked preservative film. The experiment involved three replicates at a NnFV concentration of 29 μg/mL. The distributions of mites in dish were recorded after 24 and 48 h.

Potted seedling assays: 6 peanuts and 360 adult female mites were employed. NnFV solutions (29 μg/mL) and water were sprayed on 3 peanuts as a treatment and control, respectively. Sixty adult female mites were transferred from the source culture to the freshly spouted broad leaves of one peanut after the leaves dried in the air. One treated peanut and one control peanut (CK) were put together so that the leaves of the two peanuts could touch each other. The experiment involved three replicates. Distributions of mites on the peanuts were recorded after 24, 48, and 72 h.

### 4.5. Systemic Toxicity of NnFV against T. cinnabarinus

Petiole-dip assays: The petioles of young peanut fronds were dipped into the NnFV solution (255 μg/mL) for 24 h along with the absorbent cotton covering the basal part of the petiole, and then laid on the dish (Φ 15 cm) covered on the bottom with three layers of wet filter paper. Thirty adult female mites were equally placed on the two young fronds in each slide. Then, the dish was covered with a pricked preservative film. The experiment involved three replicates. There was no proteinous acaricide as control, and Tween 20 is usually used in pesticide as an emulsifier to improve stability and homogeneity. Therefore, water containing 0.01% Tween 20 (Sinopharm Chemical Regent Co., Ltd, Shanghai, China) was used as a control. The mortality was recorded at 24, 48, and 72 h after the treatment.

Root-dip assays: The roots of peanuts were dipped into the NnFV solution (255 μg/mL) for 24 h, and picked the compound leaves for the experiment. Then, 20 *T. cinnabarinus* adult female mites were placed on the compound leaves, and the mites were put on the dish (Φ 15 cm) bottom covered with three layers of wet filter paper. The experiment involved three replicates. Water containing 0.01% Tween 20 was used as a control. The mortality was recorded at 24, 48, and 72 h after the treatment.

### 4.6. Statistical Analysis

All data were expressed as the mean ± SD of three parallel measurements. The data of acaricidal activity were analyzed by SAS v 9.3 (SAS Institute Inc, Cary, CA, USA, 2011). The toxicity regression line was evaluated with the chi-square test, and *p* > 0.05 was considered a good fitting degree of toxicity regression line. The data of repellent activity as well as systemic toxicity were analyzed by Graphpad Prism v 6.01 (GraphPad Software, San Diego, CA, USA, 2012). The significance of the differences between the means of various experimental groups was analyzed by ANOVA, followed by a *t*-test, and *p* < 0.05 was considered statistically significant.

## Figures and Tables

**Table 1:** Toxicity regression line of NnFV against *T. cinnabarinus* adult females (24 h).

Sample	NnFV	Dicofol
Linear regression equation	*Y* = 7.21 + 1.44log*x*	*Y* = 4.58 + 3.22log*x*
LC_50_ (μg/mL)	29.1	1350.9
95% Confidence interval for LC_50_ (μg/mL)	22.0–38.5	1162.5–1569.8
Toxicity index	46.42	1
χ^2^	9.8869	16.7776
*p*	0.6259	0.0523

The data were analyzed with SAS v 9.3 (SAS Institute Inc, USA), and the toxicity regression equation was tested by the chi-square test. *p* > 0.05 was considered a good fitting degree of toxicity regression equation.
